# Cortical Origins of the Flash-Lag Effect Distortions: The Influence of Retinotopic Map Architecture

**DOI:** 10.1523/JNEUROSCI.1953-25.2026

**Published:** 2026-09-16

**Authors:** Salvatore Giancani, Sandrine Chemla, Frédéric Chavane, Manuel Vidal

**Affiliations:** Institut de Neurosciences de la Timone, Aix-Marseille Université/CNRS, Marseille 13005, France

**Keywords:** flash-lag effect, retinotopy, vertical and horizontal meridian, vision, visual motion integration

## Abstract

The flash-lag effect (FLE) is an illusion whereby the position of a moving object is perceived as being offset in the direction of movement relative to a flashed object. This perceptual misalignment has been posited as a key phenomenon in explaining our ability to accurately predict the future position of moving objects, despite the delays in neuronal processing. Our working hypothesis is that the FLE is resulting from the anticipation generated by propagation of neural activity within visual cortical retinotopical maps. According to this hypothesis, the FLE should be affected by discontinuities and anisotropies of the retinotopic map architecture. Using psychophysics in humans (female ratio of 15/24), we show that the FLE is strongly affected by crossing and the direction of motion in respect to retinotopic features, such as vertical and horizontal meridians and the fovea. The specificity of how early visual cortical retinotopic maps are splitted and magnified around these features led us to suggest that the FLE distortions emerge from propagation in retinotopically organized networks, particularly V1. This work bridges the gap between human psychophysics and the known constraints of retinotopic maps layout, offering a testable framework for future studies of motion position encoding in the visual hierarchy.

## Significance Statement

The flash-lag effect (FLE) has long served as a compelling phenomenon in visual neuroscience, offering a window into how the brain enables us to interact accurately in time and space with moving objects, despite inherent processing delays. However, after decades of research, the mechanisms underlying the FLE remain largely debated, with explanations often divided among predictive coding, differential latencies, and postdiction. In our study, we proposed a new hypothesis that attributes a specific role to intracortical traveling waves and tested its behavioral consequences exploiting the known retinotopic architecture of the visual cortex.

## Introduction

The perceived position of a flashed object often lags behind the perceived position of a moving object. This phenomenon was described a century ago (Hazelhoff, 1924; Metzger, 1932), rediscovered (Mackay, 1958), and eventually termed the “flash-lag effect” (FLE; Nijhawan, 1994). The latter linked this effect to a compensatory phenomenon of the neuronal delay, named “motion extrapolation” (Khoei et al., 2017; Hogendoorn, 2020; Turner et al., 2023). The effect has been extensively studied using various stimuli condition, through the relative timing of the flash to the motion (Fröhlich, 1923; Eagleman and Sejnowski, 2007; Jancke and Erlhagen, 2010; Hubbard, 2014; Cottier et al., 2023) or different positions, motion directions, speeds, and contrasts (Schlag et al., 2000; Krekelberg and Lappe, 2001; Nagai et al., 2002; Eagleman and Sejnowski, 2007; Hubbard, 2014; Li et al., 2014). The community has debated the brain's predictive versus postdictive nature of the effect and its relationship to phenomenal awareness (Eagleman and Sejnowski, 2000, 2007; Nijhawan, 2002; Hogendoorn, 2020), with some studies speculating about a visuomotor rather than perceptual predictive role of the effect (Purushothaman et al., 1998). However, our understanding of the physiological mechanics of the phenomenon remains limited (but see Turner et al., 2023).

Numerous studies demonstrated consistent motion extrapolation in humans' EEG (Hogendoorn and Burkitt, 2018; Johnson et al., 2023; Turner et al., 2024, 2025), without pointing precisely to a particular neural structure, suggesting a cumulative effect across processing steps. Some fMRI studies demonstrated that the neural encoding of the position of a moving stimulus is shifted, with conflicting results between early and higher visual areas (Whitney et al., 2003; Maus et al., 2013; Kohler et al., 2017; Schneider et al., 2019). Evidence from animal models suggests that motion extrapolation results from multiple visual processing levels, from retinal ganglion cells (Berry et al., 1999) to the primary visual cortex in cats (Jancke, Chavane, et al., 2004a,b) and in nonhuman primates (NHP; Guo et al., 2007; Subramaniyan et al., 2018; Benvenuti et al., 2020), which involves corticocortical propagation leading to the anticipatory activity.

One possible mechanism involves connectivity between neurons with nonoverlapping receptive fields: intercortical feedback from higher areas and intracortical horizontal connectivity (Fig. 1*A*). These connections can provide information about a stimulus' arrival before the feedforward input itself, given that propagation latency along corticocortical routes (*L_H_* or *L*_FB_; Fig. 1*A*) is shorter than the overall feedforward delay (*L_H_* or *L*_FB_ < *L*_bar_). Both fast feedback myelinated fibers (1–10 m/s) from larger RF' downstream neurons and intracortical unmyelinated axons with slower propagation (∼0.3–0.5 m/s; Grinvald et al., 1994; Bringuier et al., 1999; Girard et al., 2001; Reynaud et al., 2012; Muller et al., 2014) meet this criteria for most natural moving stimuli.

Empirical evidences (Jancke et al., 2004b; Guo et al., 2007; Subramaniyan et al., 2018; Benvenuti et al., 2020) suggest that intracortical propagation within retinotopic maps (PRM) could therefore generate a reduction in neuronal latency contributing to the FLE. We refer to this as the PRM hypothesis (Fig. 1). This hypothesis yields two testable predictions: the FLE should be modulated by discontinuities of the retinotopic map, such as the vertical meridian (VM; Innocenti et al., 2022; see evidence from Benvenuti et al., 2020) and horizontal meridian (HM; Ribeiro et al., 2023), and eccentric changes in the retinocortical magnification factor (Fig. 1*B*). This builds on previous studies that have used human psychophysics to test the perceptual impact of intracortical horizontal interactions (Kapadia et al., 1995; Georges et al., 2002).

Here we used psychophysics to test these predictions at specific spatial configurations. Our results show a high FLE when motion and flash remain within the same hemifield, a sudden drop after the moving bar crosses the VM, no such drop when crossing the HM, and a modulation by eccentricity and motion direction relative to retinotopic features, all consistent with a dominant role of intracortical propagation, particularly within V1.

## Material and Methods

### Ethics statement

For each experiment of this project, participants gave a prior written consent after being informed of the methods used and their right to interrupt if they wished. Participants were compensated for each experimental protocol. This project was approved by the Comité d'éthique d'Aix-Marseille Université (reference 2014-12-3-06) and complies with the regulations described in the Declaration of Helsinki (2012).

### Subjects

We collected data from a total of 24 subjects. Of these, only two subjects participated in all four experiments, both of whom are among the authors. We analyzed data exclusively from subjects for whom the Bayesian adaptive method successfully converged (see below, Bayesian adaptive method). In Experiment 1, data from 11 subjects were collected; however, only 10 participants' data were analyzed, with a female ratio of 8/10. The mean age of the subject pool was 31.1 years (range, 24–40). In Experiments 2 and 3, data from the same 12 subjects were collected, but only 10 participants' data were analyzed, with a female ratio of 6/10. The mean age of the subject pool was 27.4 years (range, 21–41). Nearly all participants completed both experiments on the same day, except for one who was tested 2 weeks apart. Experiment 4 required a larger pool of participants: out of 16 subjects, data from 9 subjects were retained, with a female ratio of 4/9. The mean age of the subject pool was 29.8 years (range, 21–41). Due to the large number of conditions, the testing was distributed across four different sessions. The mean time difference between the first and last session was 59.6 h, with a maximum of 140 h and a minimum of 24 h. All participants had normal or corrected-to-normal vision (contact lenses were allowed, but glasses were not).

### Apparatus

Participants sat in a dark room in front of a screen, with their head movements restricted by a chin and forehead support, placing their eyes at 57 cm from the screen center. Stimuli were generated on a computer running Windows 7 operating system, with routines written in Python and using the core libraries of PsychoPy (Peirce, 2007). Visual stimuli were displayed on a Samsung TFT monitor running at a resolution of 1,680 × 1,050 and refreshed at 100 Hz (frames of 10 ms), over a uniform 50% gray background (25.8 cd/m^2^ luminance). The right eye position was monitored by a Tobii X120 video eye tracker (sampling at 120 Hz). During trials, participants were asked to look at a central fixation point represented by a small white disk (0.2° in diameter). Trials were aborted and repeated later (two trials ahead when possible) when fixation was broken, that is, when horizontal or vertical error was >0.5 and 1.2°, respectively, for at least 42 ms (five consecutive tracker frames).

### Procedure

#### General visual stimulation and task

We conducted four experiments to investigate specific aspects of the FLE. The visual stimulation always included a white fixation point on a gray background, a white bar moving along a linear trajectory at a constant speed of 6.6°/s, and a single or a pair of squares flashed for 10 ms (one frame; Fig. 2*A*, schematic of visual stimulation). The sizes and spatial layout of the bar and flashes varied across experiments and are provided in their respective sections below. The relative position of the flash and the bar was varied using a Bayesian adaptive method (see below). For Experiments 2, 3, and 4, a pair of squares were flashed on a virtual line parallel to the bar, above and below its trajectory. For Experiment 1, only one square was flashed above the bar. In all experiments, the flash was presented at the midpoint of the bar trajectories, and the task was to compare in a 2AFC the bar position relative to the flash in space. They were asked: “At the moment the flash occurred, where was the bar relative to the flash?” Responses were given either with the keyboard arrows or the numerical keypad depending on the experiment. In all trials, the bar appeared and vanished with a 100 ms fade in and out in order to minimize the saliency of transient events and subsequent involuntary eye movement. To prevent cognitive strategies while allowing sufficient response time, the reaction time (RT) was limited to a maximum of 2.5 s but with a general lower RT across participants and experiments (Figs. S2–S5). The bar disappeared either after the participant gave their answer or after having traveled a maximum of 10° (2.5 s after flashes). Trials started automatically 2 s after the previous answer or maximum RT was reached. Trials were grouped in blocks of a subset of conditions so that the participants could focus their attention to a limited area of the visual field. At the start of each block, an instruction was displayed to indicate the tested spatial area. The participant could take a short break between blocks.

#### Bayesian adaptive method

For each tested condition, an optimal Bayesian adaptive method was used to determine the tested positions of the flash relative to the bar (Kontsevich and Tyler, 1999). After each trial is answered, the method provides an estimation of both the point of subjective equality (PSE) and the standard deviation (SD) of the psychometric curve (a cumulative Gaussian), together with their respective theoretical 95% confidence intervals. Based on these, it computes the best value in the test range to be used in the next trial to maximize the gain of knowledge. This fast-converging method sampled 100 values for the flash position in the range from −1.982 and +1.32° (corresponding to a flash position that ranged from a −300 ms lag to a +200 ms lead).

#### Experiment 1: horizontal motion and flash timing relative to the VM

In Experiment 1, we tested the effect of a bar moving rightward, at the crossing or not of the VM (Fig. 2). The traveled distance before the reference flash occurred could be 1, 2, 3, and 4°. The flash appeared either 0.5° before or after the bar crossed the hemifield boundary (the VM), resulting in eight conditions: four with the bar crossing the VM and four without. The bar disappeared either after the participant answered or after traveling an additional distance of 1.5°. The bar was centered at −4° of elevation with a height of 4° and width of 0.5°. The single square flashed appeared above the bar at variable offsets (side of 0.5° and separated from the bar by 0.75°). Trials were blocked in groups of 64 in which the tested spatial position was always the same, either −0.5 or +0.5° regardless of the travel distance. Blocks were alternating with the conditions of the initial block balanced across participants.

#### Experiment 2: flash position and initial eccentricities relative to the VM

Building on Experiment 1, in Experiment 2, the FLE was measured after a fixed travel distance of 2°, but at varying eccentricities ranging from −2 to +2° in steps of 0.5° to allow for a fine characterization of the effect reduction observed when crossing the VM. This setup resulted in eight conditions, corresponding to the eight tested positions. The bar (3° in height, 0.5° in width) traveled rightward along the HM. Two squares (side of 0.5°) were flashed at the moment when the bar had traveled 2°, above and below the bar (separated by a 0.5° gap) with a variable offset. Trials were grouped in blocks of 72 in which the tested spatial positions were always in the same area, either to the left (eccentricities −2 to −1°) or to the center (−0.5 to +0.5°) or to the right (+1 to +2°). The order of the first three blocks was randomized across participants and repeated for the remaining blocks of the experiment.

#### Experiment 3: vertical motion and flash timing relative to the HM

Similarly to Experiment 2, in Experiment 3 the FLE was measured after a fixed travel distance of 2°, at varying eccentricities (−2, −0.5, +0.5, and +2°) to allow a characterization of possible effects given by crossing of the HM. The bar (3° in height, 0.5° in width) traveled along the VM, both in upward and downward directions. Similarly to the previous experiment, this setup resulted in eight conditions: four tested positions and two motion directions. Two squares (side of 0.5°) were flashed at the moment when the bar had traveled 2° to the right and left of the bar (separated by a 0.5° gap) with a variable offset. Trials were grouped in blocks of 64 in which the tested spatial positions were always in the same area, either above (eccentricities of −2, −0.5°) or below (+0.5 and +2°) regardless of motion direction. Blocks were alternating with the conditions of the initial block balanced across participants.

#### Experiment 4: flash position proximity to fovea, VM, and HM

To investigate the effects of proximity to the fovea, VM, and HM and potential interactions between these factors, we designed Experiment 4. In this experiment, the moving bar could have one of eight directions (from 0 to 360° in steps of 45°). The bar (1.8° in height, 0.3° in width) moved toward four different tested positions: on the fovea ([0°, 0°]), on the VM ([0°, −2°]), on the HM ([2°, 0°]), and away from the fovea, VM, and HM ([2°, −2°]). After traveling a visual distance of 2°, the bar reached the tested position, and two squares were flashed (side of 0.3°) at each ending of the bar (separated by a 0.5° gap) with a variable offset. The experiment has a total amount of 32 conditions. Trials were grouped in blocks of 32 in which the tested spatial position was always the same (e.g., [0, 0], [+2, 0], [0, −2], or [+2, −2]) so that attention was spatially focused on the same location, and the motion direction was either cardinal (0, ±90, and 180°) or oblique (±45 or ±135°). Answers were given using the numerical keypad of the keyboard, and participants were instructed to keep four fingers on the corresponding directions (8/2 and 4/6 for cardinal or 1/9 and 7/3 for oblique motion directions, respectively). At the start of each block, an instruction was displayed to the participant with a scheme indicating the tested spatial location, the four possible motion directions, and its corresponding keys. In each block, conditions were repeated eight times. The order of blocks was determined so that the two motion directions (cardinal and oblique) in a given spatial location were tested successively, and the spatial location tested was assigned to subject numbers using a 4 × 4 Latin square design to cancel for possible order biases in the results. Each experimental session contained 24 blocks, for a subtotal of 768 trials. The experiment contained four sessions, for a total of 3,072 trials including 96 repetitions of each condition.

### Data analysis

#### Nonlinear regressor model

To analyze the responses to a moving bar stimulus across different positions in the visual field (as described for Experiment 4), we developed a set of parametric models based on the spatial configuration and movement directions.

For each of the four conditions (foveal, centered on VM, centered on HM, away from the fovea, HM, and VM), we modeled the perceptual responses as a function of the stimulus direction using nonlinear regression functions (Fig. 5*C*–*E*). The general form of the models incorporates a combination of cosine and sine components, capturing the horizontal and vertical motion contributions. The principal rationale is to compute the variation of trajectory to a retinotopic feature [being the two cardinal meridians (horizontal H and vertical V) and the fovea-fixation point ( F)]. These components are equally weighted by the eccentricity of the trajectory, represented by an absolute distance between the fixation point and the mean point of the bar trajectory: the eccentricity is indicated as ecc, with trajectory length indicated by a (Fig. 5*D*).

We formalize the model as follows:

model(θ,c,a)=q+e⋅ecc(θ,c,a)⋆D(θ,c,a),

with q as baseline and D(θ,c,a) distances from HM, VM, and fovea as follows:

D(θ,c,a)=f⋅F(θ,c,a)+h⋅H(θ,c,a)+v⋅V(θ,c,a).

The independent variable *θ* corresponds to the motion direction. It corresponds to the eight directions between 0 and 360°, stepping 45°. c is the center of stimulus presentation $\left(x_{0},y_{0}\right)$: it corresponds to the position when the flashing reference occurs, and we impose that at this point the bar has traveled for a length a. The model finds the set of values q, e, f, h, and v that better approximate the results obtained from subjects in Experiment 4. Below the components F(θ,c,a), H(θ,c,a), V(θ,c,a), and ecc(θ,c,a) are described. For the four positions employed in Experiment 4, we have for foveal ergo c=(0,0) as follows:

$$\begin{array}{rl} F_{\left(0,0\right)}(\theta ,a)\;= & \sqrt{a^{2}\sin^{2}\theta +a^{2}\cos^{2}\theta }=a\\ H_{\left(0,0\right)}(\theta ,a)\;= & |a\sin\;\theta |\\ V_{\left(0,0\right)}(\theta ,a)\;= & |a\cos\;\theta |\\ \mathrm{ec}c_{\left(0,0\right)}(\theta ,a)\;= & \sqrt{\frac{a^{2}}{4}\sin^{2}\theta +\frac{a^{2}}{4}\cos^{2}\theta }=\frac{a}{2}. \end{array}$$

For centered on VM, ergo c=(0,−d):

$$\begin{array}{rl} F_{\left(0,-d\right)}(\theta ,a)\;= & \sqrt{{\left(d-a\sin\;(\theta +\pi )\right)}^{2}+{\left(a\cos\;(\theta +\pi )\right)}^{2}}-d\\ H_{\left(0,-d\right)}(\theta ,a)\;= & (d-a\sin\;(\theta +\pi )-d)=a\sin\;\theta \\ V_{\left(0,-d\right)}(\theta ,a)\;= & |a\cos\;\theta |\\ \mathrm{ec}c_{\left(0,-d\right)}(\theta ,a)\;= & \sqrt{{\left(d-\frac{a}{2}\sin\;(\theta +\pi )\right)}^{2}+{\left(\frac{a}{2}\cos\;(\theta +\pi )\right)}^{2}}. \end{array}$$

For centered on HM, ergo c=(d,0):

$$\begin{array}{rl} F_{\left(d,0\right)}(\theta ,a)\;= & \sqrt{{\left(d+a\cos\;(\theta +\pi )\right)}^{2}+{\left(a\sin\;(\theta +\pi )\right)}^{2}}-d\\ H_{\left(d,0\right)}(\theta ,a)\;= & |a\sin\;\theta |\\ V_{\left(d,0\right)}(\theta ,a)\;= & d+a\cos\;(\theta +\pi )-d=-a\cos\;\theta \\ \mathrm{ec}c_{\left(d,0\right)}(\theta ,a)\;= & \sqrt{{\left(d+\frac{a}{2}\cos\;(\theta +\pi )\right)}^{2}+{\left(\frac{a}{2}\sin\;(\theta +\pi )\right)}^{2}}. \end{array}$$

For the case away from the fovea, HM, and VM, ergo c=(d,−d):

$$\begin{array}{rl} & F_{\left(d,-d\right)}(\theta ,a)\;=\;\sqrt{{\left(d-a\sin\;(\theta +\pi )\right)}^{2}+{\left(d+a\cos\;(\theta +\pi )\right)}^{2}}-\sqrt{2}d\\ & H_{\left(d,-d\right)}(\theta ,a)\;=\;d-a\sin\;(\theta +\pi )-d\;=\;a\sin\;\theta \\ & V_{\left(d,-d\right)}(\theta ,a)\;=\;d+a\cos\;(\theta +\pi )-d\;=\;-a\cos\;\theta \\ & \mathrm{ec}c_{\left(d,-d\right)}(\theta ,a)\;=\;\sqrt{{\left(d-\frac{a}{2}\sin\;(\theta +\pi )\right)}^{2}+{\left(d+\frac{a}{2}\cos\;(\theta +\pi )\right)}^{2}}. \end{array}$$

For all the nonfoveal positions, a component π is added to the θs, changing the sign of the equations: this is due to the assumption that the biggest FLE is associated with foveo-petal directions.

The margin of error of the fit is computed as the half-width of the confidence interval (Fig. 5*A*, light blue shaded area), with a 95% of confidence level.

The code for fitting analysis and mathematical implementation of the equations is available at the repo https://github.com/SaGiancani/model-fle.

#### Permutation test on model fitting

We systematically shuffled the data's relationship to the stimulus features (random attribution of position and direction) to assess the model's statistical significance by comparing the fit quality—fit *r*-squared (*R*^2^) and rooted mean squared error (rMSE)—for shuffled data and actual data. Data points from the psychophysical data of Experiment 4, at a single-subject level and on the group average, were shuffled across motion direction and position and then fitted according to our geometrical model. The process was repeated 1,500 times. *P* values were calculated for each subject and for the overall average across subject distributions to evaluate statistical significance. Code and results for the permutation analysis of model fitting are available at https://github.com/SaGiancani/model-fle.

## Results

Our working hypothesis is that the FLE arises from the intracortical propagation of information within visual retinotopic maps, a framework we term the PRM hypothesis. According to this view, the FLE should be modulated by both discontinuities in the retinotopic layout and changes in cortical magnification, including its anisotropic distribution across vertical and horizontal dimensions (Himmelberg et al., 2023). These structural factors are expected to influence our ability to represent the position of moving stimuli ahead of their actual locations (Fig. 1).

**Figure 1.**
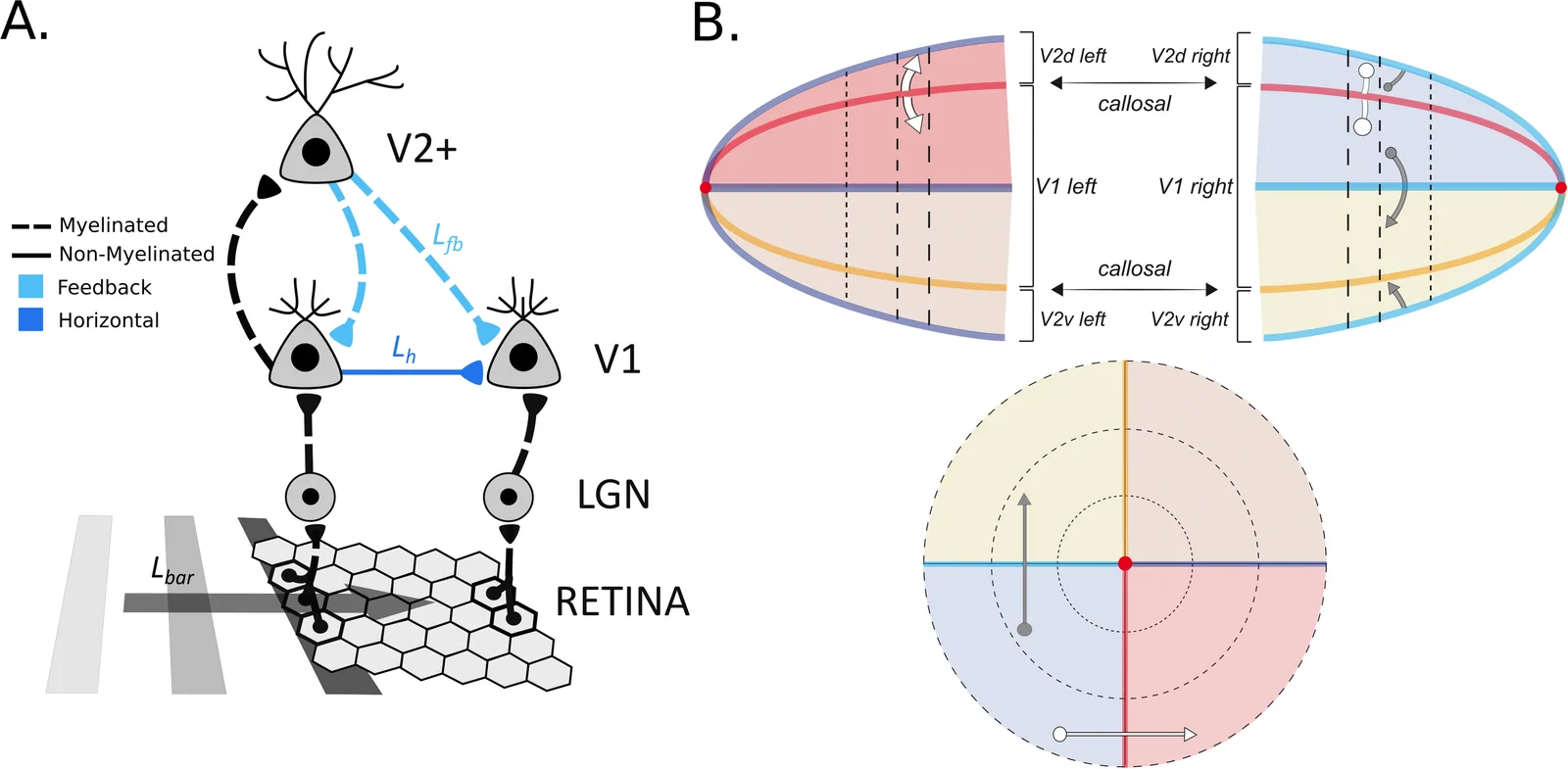
Illustration of the PRM hypothesis. ***A***, Schematic of the propagation of information within a retinotopic map. A moving bar activates the retina and takes some time to reach the trajectory endpoint (*L*_bar_, gray). This is followed by a sequence of feedforward activations, mediated by myelinated fibers (black dashed lines), to the lateral geniculate nucleus (LGN) and the primary visual area (V1). At this level, two different but concurrent types of propagation can occur: the information passes to higher visual areas (V2+) through feedforward myelinated fibers and returns to V1 with fast and myelinated feedback connections (light blue dashed lines) with high spatial divergence and reaching the representation of the trajectory endpoint in V1 with latency *L*_fb_ (cyan); in parallel, the information propagates within V1 through slower horizontal connectivity (blue line) and activates V1 representation of the trajectory endpoint with a latency *L_h_*. Both dynamics may induce a drop in temporal latency if *L_h_* or *L*_fb_ < *L*_bar_, mediating an early activation of cortical areas otherwise still untouched by the feedforward flow of the bar's movement. ***B***, Schematic of the visual field with color-coded retinotopic features and quadrants. In the lower panel, a representation of the visual field is shown, with evenly spaced dashed isoeccentric circular lines and bold colored isopolar lines, corresponding to horizontal (HM, cyan and purple for left and right HM, respectively) and vertical meridians (VM, orange and red for upper and lower VM, respectively). The crossing of HM and VM represents the fovea (red dot). The lower hemifields are represented in light red and light blue (respectively, right and left), and the upper hemifields are represented in light orange and light yellow (respectively, right and left). A horizontal trajectory (white arrow) crosses the lower VM, while a vertical trajectory (gray arrow) crosses the left HM. In the top panel, flat maps of V1 and V2 are displayed. The dashed isoeccentric circular lines are now represented as vertical lines and no longer equispaced due to the rescaling caused by the cortical magnification factor. At the outermost part of the two schematized hemispheres is the foveal representation (the red dots). Right and left hemispheres are separated along the V1/V2 border in the dorsal and ventral positions for lower (red) and upper (orange) VM, respectively. Neurons close to the border are communicating through callosal connections (black arrows) that bind the right and left hemifields through the VM. It follows that the cortical representation of the white trajectory crossing the lower VM appears split along the cortical representation of the lower VM. The representation of the arrow's head and tail is located on the dorsal left and right hemifield, both for V1 and V2. The cortical representation of V2+ is splitted into quadrants, with left and right hemifield represented contralaterally and upper and lower hemifield represented in dorsal and ventral V2. As a result, the V2 cortical representation of the vertical gray trajectory that crosses the HM in the right hemifield has its head and tail represented in the ventral and dorsal parts of V2, respectively, generating a continuous activation in V1.

To test this hypothesis, we conducted a series of experiments in which we measured the strength of the FLE at different spatial locations of the visual field and for various motion directions (e.g., horizontal, vertical, or oblique). Participants had to compare the perceived position of a bar translating at constant speed relative to a briefly flashed single/pair of squares aligned with the bar's orientation (Kanai et al., 2004; Wang et al., 2021; Amadeo et al., 2022).

### Experiment 1

In a first experiment, we tested how the FLE changes when crossing the VM and whether its strength depends on the traveled distance. Participants were presented with bars translating horizontally below the HM (right-to-left, speed of 6.6°/s and distance ranging from 1 to 4°) before a square was flashed above the bar with a given horizontal offset. The task was to judge whether the bar was perceived to the right or to the left of the square (Fig. 2*A*). The flash offset was varied using a Bayesian adaptive method to estimate the participants point of subjective equivalence and the standard deviation (PSE and SD), i.e., the position of the square perceived as horizontally aligned with the bar and its precision. We compared the FLE observed in two different test positions (the spatial location of the bar at the time the square is flashed), either 0.5° before the VM (same hemifield; Fig. 2*B*, blue) or 0.5° after the VM (crossing hemifield, red). The first result was a dramatic global reduction of 0.28° of the FLE measured just after crossing the VM compared with just before (0.45 vs 0.17°, *t*_(9)_ = −8.04, *p* < 0.0001), and this reduction was consistent across the four tested traveled distances (all *p* < 0.001). The distance traveled had no influence: motion past history did not increase nor decrease the FLE observed. We conducted two control experiments to test if these findings depend on the motion direction (i.e., different FLE for bars moving leftward than rightward) or on the tested hemifield (i.e., different FLE for bars moving rightward in the left hemifield and leftward in the right hemifield). We found a similar reduction of the FLE after crossing the VM (0.39 vs 0.14°, *t*_(7)_ = −3.92, *p* < 0.005) when reversing the motion direction, and no difference between hemifields (0.44 vs 0.45°, *t*_(9)_ = 0.23, *p* = 0.8) within the same range of distances (Fig. S1). As a consequence, in the next experiment, we kept only rightward motion. Results for single subjects are shown in Figure S2.

**Figure 2.**
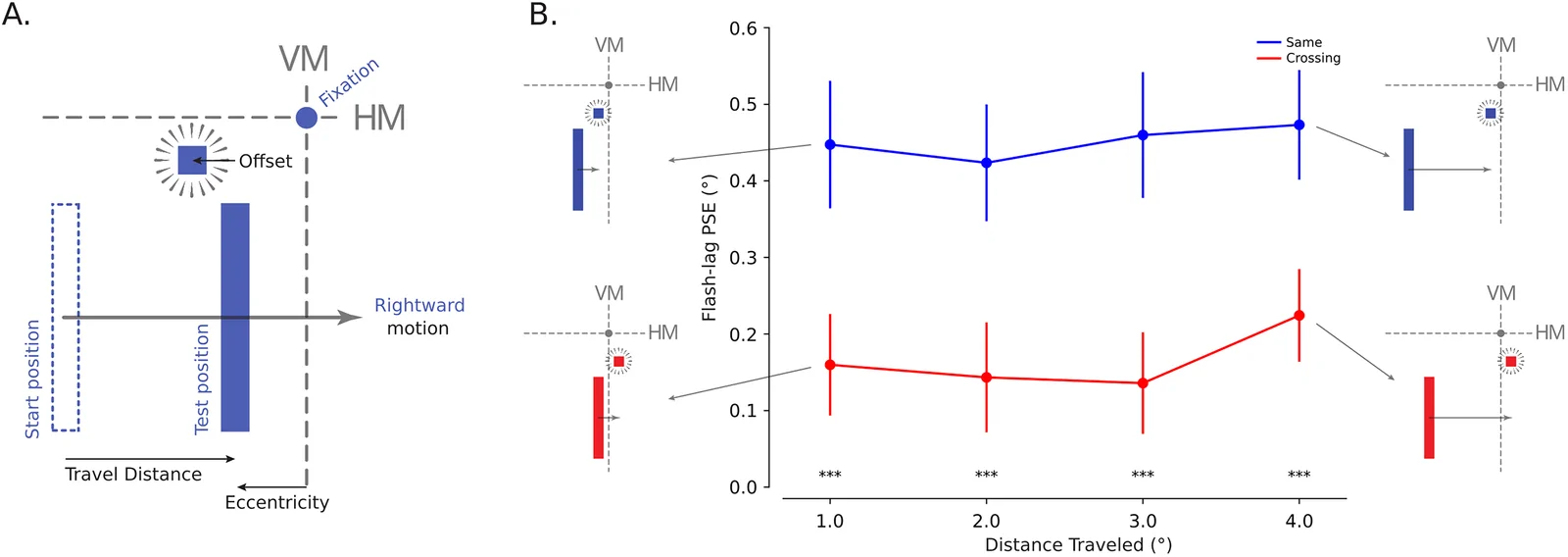
Experiment 1: The FLE is selective to the hemifield where the motion occurs. ***A***, Schematic of the flash-lag visual task and glossary of the manipulated experimental factors. The bar is moving rightward, with the start position in the lower-left hemifield. The distance between the starting position and the test position corresponds to the traveled distance, with both starting and test positions that can have different eccentricities. The square is flashed with an offset relative to test position determined by a Bayesian adaptive method (see Materials and Methods). The dashed lines represent the VM and HM. The small blue disk represents the center (and fixation point). ***B***, The strength of the FLE in degrees, measured either 0.5° before the VM (same hemifield, blue dots) or 0.5° after the VM (crossing hemifield, red dots), is plotted as a function of the distance traveled. Dots and error bars represent PSE ± standard errors across subjects. Illustrations of the stimuli used for both conditions in the 1 and 4° distance are shown on the side.

### Experiment 2

The second experiment was designed to characterize precisely the drop of the FLE observed after crossing the VM. In this experiment, the bar moved along the HM for a distance of 2° when a pair of squares—above and below the bar—was flashed with a given horizontal offset. The horizontal eccentricity of the test position ranged from −2 to +2° in steps of 0.5° (Fig. 3), so that the bar motion was either going toward (VM-petal, −2 to −0.5°, blue dots) or away from the VM (VM-fugal, +0.5 to +2°, red and green dots) at the moment of the flash. Consistent with the findings of the first experiment, the mean FLE measured when the bar remained in the same hemifield (−2 to −0.5°, blue) was significantly larger than when crossing the VM (+0.5 to +2°, red and green), with 0.38 and 0.05°, respectively (*t*_(9)_ = 6.59, *p* < 0.0001). Within the VM zone (−0.5 to +0.5°), the FLE dropped linearly with a slope of −0.34 degree of PSE per degree of visual eccentricity (*p* < 0.03). These results reveal that the drastic reduction occurs within a sharp range of tested eccentricities. Importantly, there was only a limited rebuild of the FLE after crossing the VM, with small, yet significant, FLE increase at +1° (*p* < 0.05), followed by a reduction at larger eccentricities with a FLE not significantly different than 0 at 1.5 and 2°. Seven participants showed a gradual but incomplete rebuild of the FLE, and four participants showed a decrease leading to negative FLE (see individual results in Fig. S3). It should be noted that at large positive eccentricities (VM-fugal motion), the response precision decreases, as indicated by increasing SD of the PSE across participants (Fig. S3). This suggests an important difference in the modulation of FLE for motion toward or away from the VM (VM-petal vs VM-fugal) that is strongly dependent on interindividual differences.

**Figure 3.**
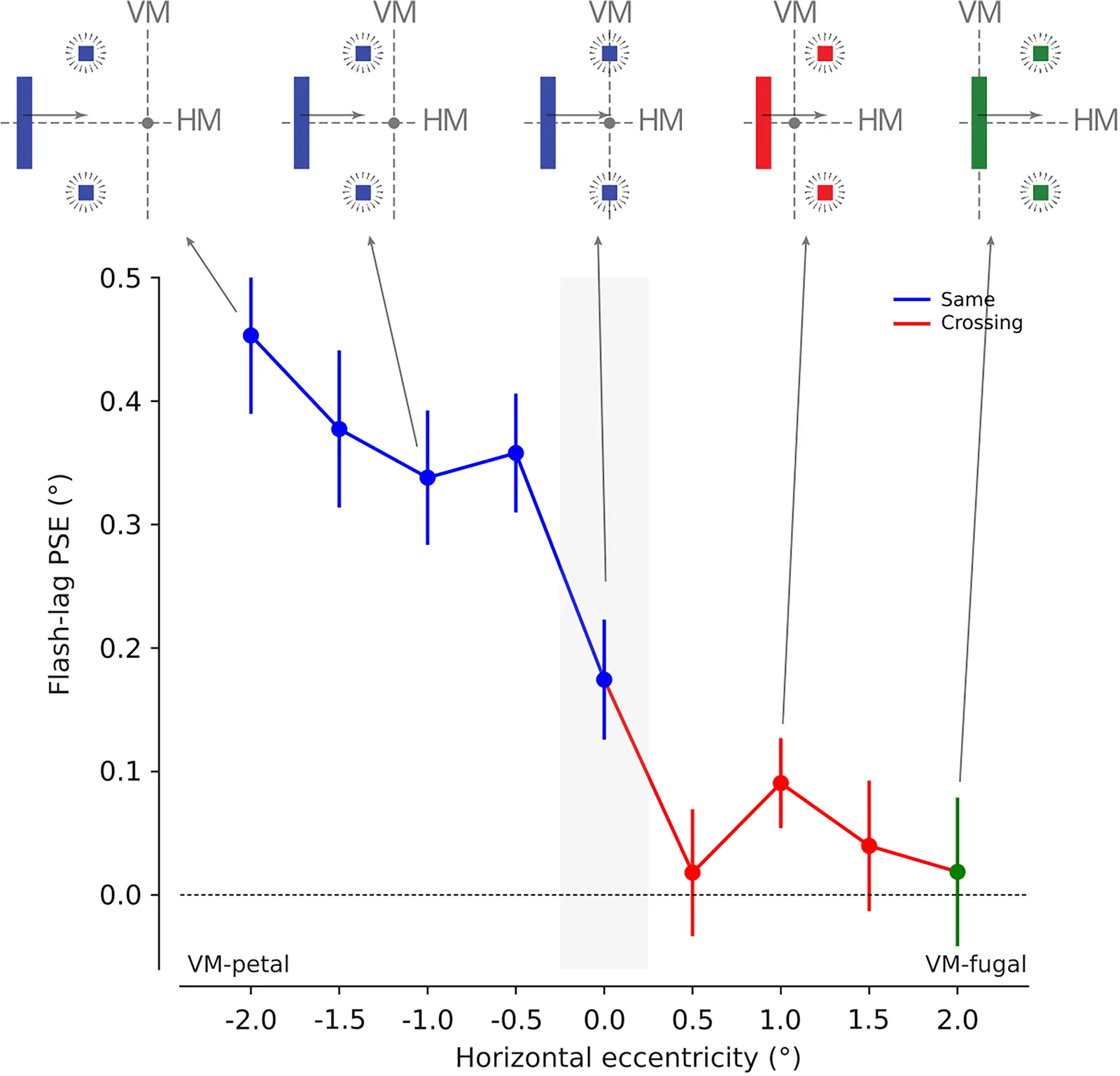
Experiment 2: The FLE drops very rapidly when crossing the VM. The strength of the FLE in degrees as a function of the horizontal eccentricity of the tested position. We distinguished three situations (represented by the three colors): when the bar moves toward the VM (VM-petal direction) and the flashing squares occurs before crossing the VM (in blue), when the bar crosses the VM (VM-petal then -fugal directions) before the flash occurs (in red) and finally when the bar starts at the fovea and moves entirely in VM-fugal direction (in green). Spatially coherent illustrations of the stimuli for selected conditions are visible on the top of the figure. The gray shaded area ∼0° eccentricity highlights the range where the 0.5° bar width overlaps the VM. The position of the flashes relative to the tested position was determined by a Bayesian adaptive method.

### Experiment 3

To further test the role of anatomical discontinuities of retinotopic maps, the third experiment was designed to characterize how the FLE evolves after crossing the HM. In this experiment, a horizontal bar was translated vertically along the VM, either moving upward or downward, for a distance of 2° when a pair of squares—right and left of the bar—was flashed at a given vertical offset. The vertical eccentricity of the test position defining the conditions is given relative to the motion direction, so that we can compare the upward and downward directions seamlessly. Four vertical eccentricities relative to motion were compared: −2, −0.5, +0.5, and +2° (Fig. 4). At the moment of the flash, the bar was moving either toward the HM for negative eccentricities (HM-petal, blue) or away from the HM for positive eccentricities (HM-fugal, red and green). Contrarily to what was reported when crossing the VM (Experiment 1 and 2), at 0.5° eccentricity, the FLE was higher when the bar crossed the HM (HM-fugal in red) than when it remained in the same hemifield (HM-petal in blue), for both upward (0.19 vs 0.06°, *t*_(9)_ = 3.29, *p* < 0.01) and downward motions (0.34 vs 0.16°, *t*_(9)_ = 3.26, *p* < 0.01). At 2° eccentricity, the FLE was not different between HM-petal and HM-fugal whether moving upward or downward (*p* > 0.5 for both). Finally, upward and downward motion directions generated significantly different FLE at +0.5° (*t*_(9)_ = 3.59, *p* < 0.006) and nearly at −0.5° (*t*_(9)_ = 2.02, *p* = 0.075). Hence, contrary to the VM crossing and at small eccentricities, the FLE was selective to the motion direction, being stronger for bars moving downward compared with upward. Individual results are shown in Figure S4.

**Figure 4.**
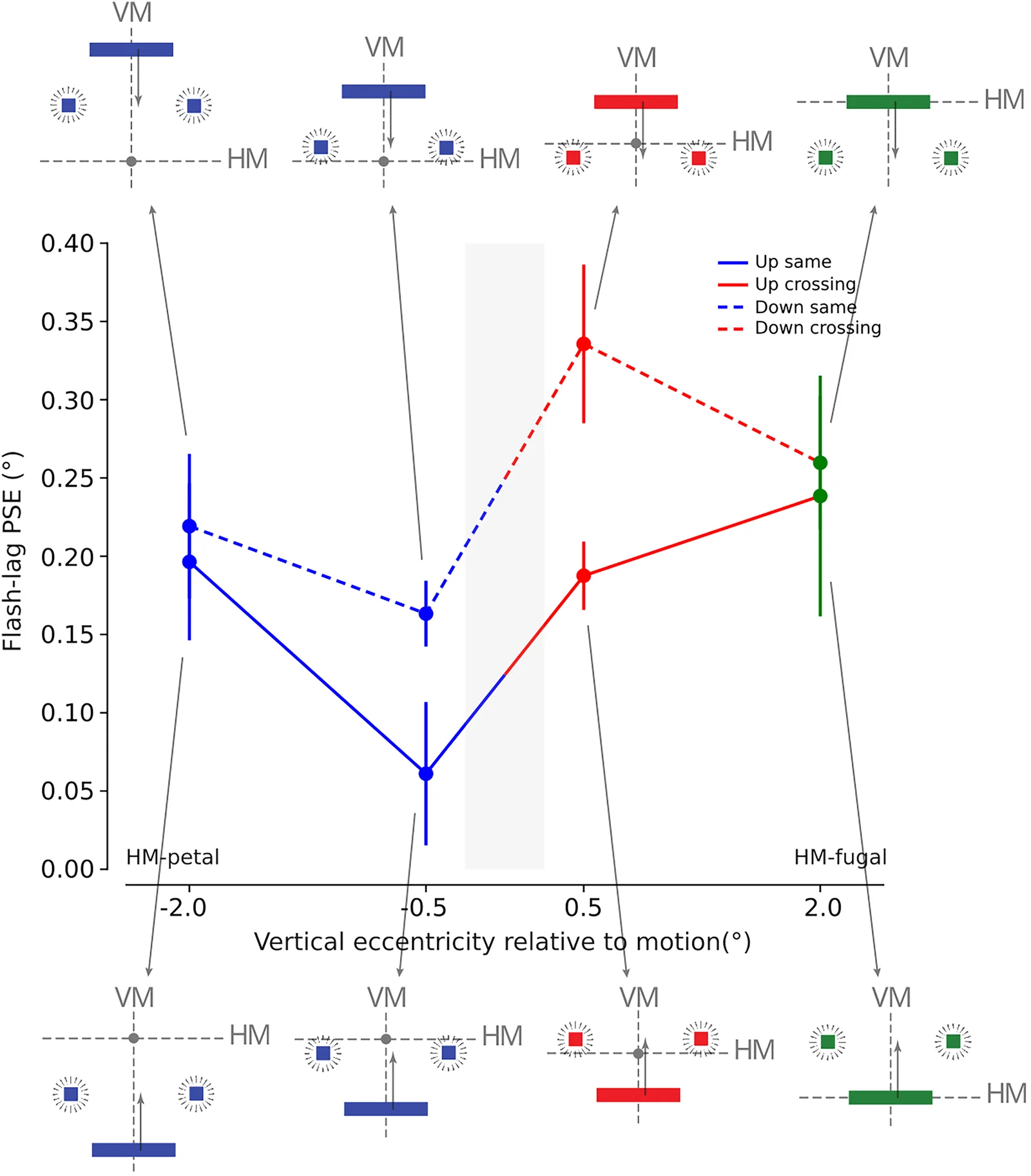
Experiment 3: The FLE around the HM. The strength of the FLE in degrees as a function of the vertical eccentricity relative to motion of the tested position. The bar could move upward (solid line) or downward (dashed line). We distinguished three situations: when the bar moves toward the HM and the flashing test occurs before crossing (HM-petal only, in blue); the flashing test occurs after the bar crosses the HM (HM-petal then HM-fugal, in red) and finally when the bar starts to move from fovea and then flashing test occurs (HM-fugal only, in green). Illustrations of the stimuli are visible on the top of the figure. The gray shaded area around 0° eccentricity highlights the range where the 0.5° bar width overlaps the HM. The position of the flashes relative to the tested position was determined by a Bayesian adaptive method.

From the three previous experiments, we came to the conclusion that other retinotopic features could affect the FLE. First, the FLE reduction was stronger in Experiment 2 compared with Experiment 1. Given that the main difference between these experiments was that only the VM was crossed in Experiment 1, whereas both VM and fovea were crossed in Experiment 2, it suggests that the fovea could also play a role. Furthermore, in Experiment 2, we noticed that the FLE was much stronger for motion toward the VM (blue) than away from the VM (red and green). In Experiment 3, crossing the HM modulates FLE differently than crossing the VM, pointing to the distinct functional role of the HM.

### Experiment 4

Based on the observations in Experiment 2 and 3, the fourth experiment was designed to fully disentangle the physiological biases on the FLE introduced by visual motion toward and away from the fovea, the VM, or the HM. In this experiment, we measured the FLE in four test positions of the visual field for eight motion directions (0, 45, 90, 135, 180, 225, 270, 315°) resulting in a total of 32 conditions. The test position was either foveal ([0°, 0°]), on the VM but away from the fovea ([0°, −2°]), on the HM but away from the fovea ([2°, 0°]), or away from the HM, VM, and fovea ([+2°, −2°]). As in previous experiments, the bar moved 2° before two squares appeared above and below the bar (relative to its motion), at a variable offset (see Materials and Methods, Bayesian adaptive method). Importantly, in this experiment, the bar did not cross VM, HM, or fovea (see Fig. 5*C* for a visual schematic) before the flash occurred. A first clear effect is that of the tested position in the visual field, with the conditions at position [2°, −2°] showing a larger FLE modulation than other positions (Fig. 5*A*). A second effect is that of motion direction, except for the foveal position, [0°, 0°] where the FLE remains around 0.2° for all eight directions. In all the other positions ([+2°, −2°], [0°, −2°], and [+2°, 0°]), we observe an increase of FLE in foveo-petal versus foveo-fugal motions, with values near 0.35° for foveo-petal and slightly below 0.2° for foveo-fugal. This difference is only significant at position [2°, −2°] (0.48 vs 0.08°, *t*_(9)_ = 3.73, *p* = 0.006). Consistently, the diagonal motion directions at positions [2°, −2°] and [+2°, 0°] also show a stronger FLE when they contain a motion-component toward the VM (directions 135 and 225°) compared with away from the VM (direction 45 and 315°).

**Figure 5.**
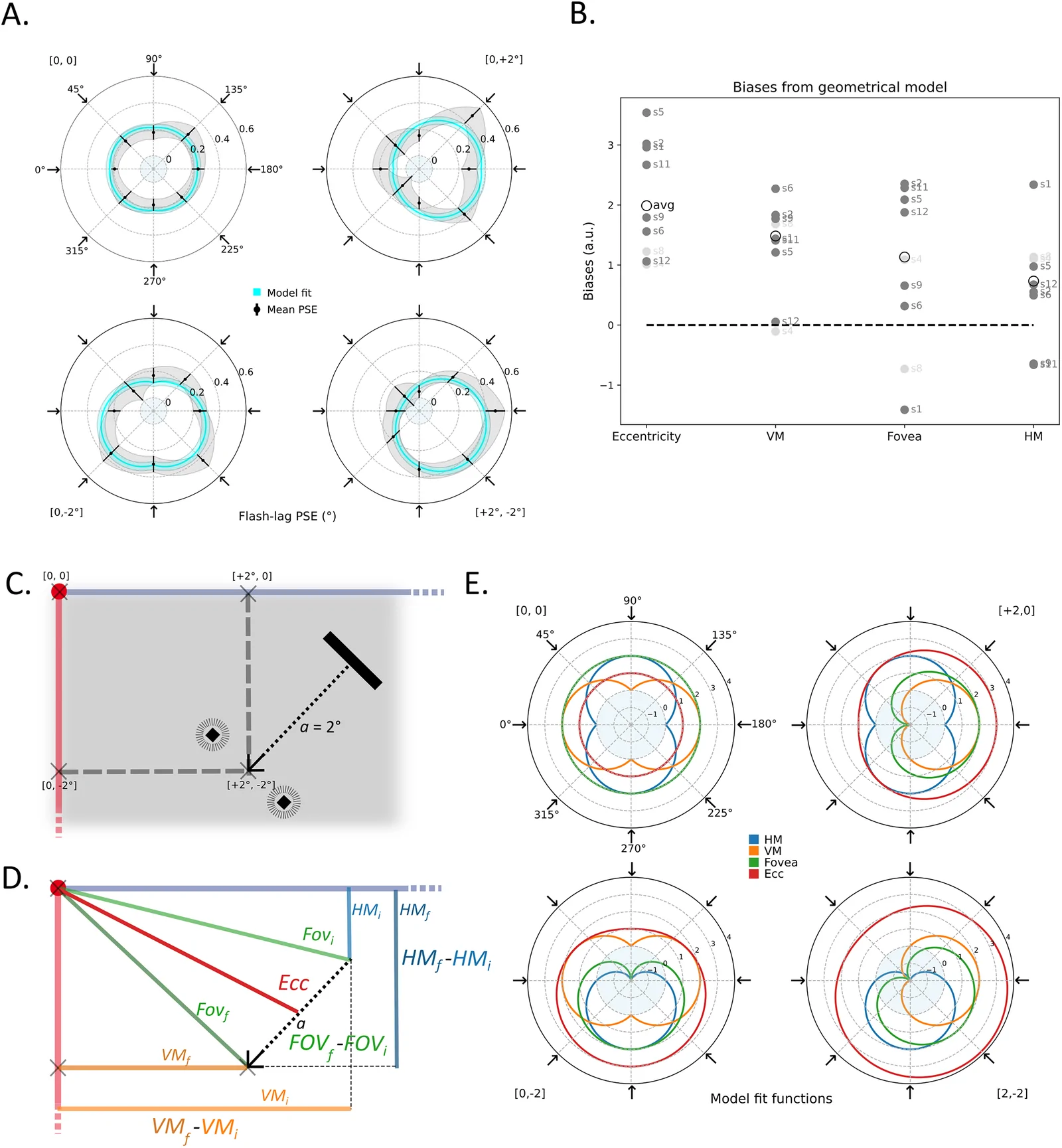
Experiment 4: Disentangling the effects of motion relative to retinotopic features on FLE through a geometrical model. ***A***, Polar representation of the strength of the FLE in degrees (distance to center) as a function of the bar motion direction and value of bias of the geometrical model (angle of the plot). Each of the polar plots corresponds to one of the four positions where the FLE was measured: foveal [0°, 0°], on the VM and away from the fovea [0°, −2°], on the HM and away from the fovea [2°, 0°], or away from the HM, the VM, and the fovea [2°, −2°]. The bar could move in eight different directions systematically shown by the arrows at the most external part of each polar plot (with the corresponding direction angle reported in the first top-left polar plot). Dots and error bars indicate the mean ± standard error of PSE across subjects; the gray shaded area showing interpolated values between tested directions. The light blue shaded disks in the center of polar plots represent FLE negative values (−0.1 to 0°). The light blue thick line represents the fitted model on the averaged data for each position, while the shaded area represents the 95% confidence interval of the model fit. ***B***, The figure shows values of the four biases of the geometrical model: eccentricity, VM, fovea, and HM. Dark gray dots represent individual subjects, labeled by subject number for which the fitting model was reported significant (with permutation test). Subjects 4 and 8 for which the fit does not perform better than for random sorted data are reported in light gray. The empty black dots show the averages computed across all subjects, used for the model fit representation of Panel ***A*** (the light blue thick lines). ***C***, Schematic of the stimulus used in Experiment 4. Bars could move in eight possible directions (illustration with a bar moving in the 135° direction). Bars traveled a distance of 2° before two flashing targets appear above and below at a certain offset, determined by a Bayesian adaptive method. Bars could travel toward four possible positions: [0°, 0°], [0°, −2°], [+2°, 0°], [+2°, −2°]. ***D***, In the situation illustrated in panel ***A***, the geometrical model is based on the relative distances to each retinotopic features: horizontal meridian (HM in blue), vertical meridian (VM in orange), and fovea (Fov in green), between final (subscript *f*) and starting (subscript *i*) positions. The eccentricity (Ecc in red) measures the foveal distance to the middle of the trajectory. The same color convention as in Figure 1*B* was used for panels ***A*** and ***B***, fovea being represented at [0°,0°] with a red dot and right HM and lower VM being represented in violet and light red, respectively. ***E***, Polar plots of the geometrical models at each tested position and direction for each of the biases.

VM-fugal and VM-petal motion (0 and 180° directions, respectively) at position [2°, 0°] are not significantly different (0.19 vs 0.28°, *t*_(9)_ = 1.11, *p* = 0.30). Similarly, no significant difference was reported for VM-fugal motion (direction 0°) at position [2°, 0°] and VM-petal motion (direction 0°) at position [0°, 0°] (0.19° vs 0.19°, *t*_(9)_ = 0.03, *p* = 0.98). However, the three conditions—VM-fugal and petal motion at position [2°, 0°] (directions 0° and 180°, respectively) and VM-petal motion at position [0°, 0°] (direction 0°) in Experiment 2 (tested positions −2, 0, and +2°; see the first and last blue dots and the green dot in Fig. 3)—lead to a significant difference, with a stronger FLE for HM. These discrepancies may reflect differences in the participant pool, as supported by the high interindividual variability in Figure 5*A* (apparent in Fig. 5*B*; see next chapter) for this condition or in the specific grouping of conditions for the two experiments (see Materials and Methods). Interestingly, our model (detailed in the next chapter) predicts such a difference with a lower VM-fugal FLE than a VM-petal FLE (see the blue line at [0°, 0°], [2°, 0°] in Fig. 5*A*).

The effect of moving toward or away from the HM is weaker compared with the other retinotopic features, with a stronger FLE for HM-petal conditions than HM-fugal conditions observed only for positions far from the HM: at [0°, −2°] (0.30 vs 0.17°, *t*_(9)_ = 3.45, *p* = 0.009) and [+2°, −2°] (0.34 vs 0.05°, *t*_(9)_ = 3.45, *p* = 0.002). The conditions +2° with downward motion (HM-fugal) and −2° with upward motion (HM-petal) of Experiment 3 are similar to the 270° direction (HM-fugal) and 90° direction (HM-petal) at position [0°, −2°] of Experiment 4. The trend observed in Experiment 3 between HM-petal and HM-fugal (0.26 vs 0.196°, *p* = 0.23) became significant in Experiment 4 (0.30 vs 0.17°, *t*_(9)_ = 3.45, *p* = 0.009).

#### Geometrical modeling of physiological biases

All tested conditions of this experiment combine physiological biases related to three retinotopic features (fovea, VM, and HM) and eccentricity. In order to disentangle, quantify, and compare the respective effects on the FLE of visual motion toward (petal conditions) or away from (fugal conditions) these retinotopic features, we developed geometrical models of each of these biases and fitted their respective weights to the global effect observed (see Materials and Methods). In this model, we took into account the positive/negative variation of distance to these retinotopic features along the motion trajectories, assuming that the interaction between the three retinotopic features combined linearly (Fig. 5*D*). The fitted model (Fig. 5*A*, light blue curves) captured the overall trend of the group-averaged data (*R*^2^ = 0.768), with a permutation test confirming the statistical significance of the fit (*p* = 0; see Materials and Method). We define the null hypothesis as the case where the data has no systematic relationship with the stimulus features, namely, position and motion direction. Consequently, the model's fit quality (*R*^2^, rMSE) should be no better than expected from data in which these relationships have been randomly shuffled (see Materials and Methods for more details).

The permutation test was also applied at the single-subject level, rejecting the null hypothesis for seven out of nine subjects: only two subjects had *p* values between 0.01 and 0.05 (sub12: *R*^2^ = 0.234, *p* = 0.047; sub1: *R*^2^ = 0.322, *p* = 0.015), while the remaining five had *R*^2^ > 0.385 and *p* < 0.01. The model allowed quantifying for each individual subject as well as for the group average, how motion direction (toward or away from) relative to each retinotopic features (fovea, VM, and HM), and how eccentricity can bias the FLE. Eccentricity gave the strongest bias on the group average (1.989), indicating that the FLE increases with eccentricity independently from its direction. For directional biases (toward or away from), by convention, a positive bias indicates an increased FLE in petal versus fugal conditions. The strongest directional bias was observed for VM (1.484) and then fovea (1.133), and the smallest was for HM (0.732). Analyzing these biases for each subject (Fig. 5*B*), we found that VM and foveal biases are both stronger than HM biases in six subjects out of nine. Overall, there are no clear correlations between any of the biases. Data of a typical participant is shown in Figure S5.

## Discussion

In this study, we tested the predictions of the PRM hypothesis, which proposes that the FLE arises from activity propagation within retinotopic cortical maps. Under this framework, discontinuities and deformations in retinotopic maps will modulate the effect. To evaluate these predictions, we conducted a series of psychophysical experiments in humans.

We found a marked reduction of the FLE when the moving stimulus crossed the VM, independent of motion direction and trajectory length. This reduction occurred within <1° of visual angle across the VM. Importantly, the FLE did not fully recover after crossing: values remained lower for VM-fugal than VM-petal motion. In contrast, the FLE increased upon crossing the HM at low eccentricity (0.5°) for both upward and downward trajectories, with larger values for downward motion. Lastly, we designed an experiment to disentangle the effects of eccentricity, motion direction, and proximity to the fovea, VM, and HM. The FLE increased with eccentricity and for motion toward retinotopic features (petal) relative to motion away (fugal). Hence, the FLE reflects multiple independent biases, dominated by eccentricity and VM-related factors, with smaller contributions from foveal and HM biases. This is the first study that characterizes how the FLE is modulated by crossings of retinotopic features, motion direction, and eccentricity. A key strength was the high-resolution sampling of central vision, enabled by using a Bayesian adaptive procedure.

### Comparison with literature

Several studies have investigated the influence of retinotopic boundaries on other positional visual illusions. Liu et al. (2018) showed that illusory position shifts were reduced when motion does not cross the VM or HM, attributing this effect to interareal discontinuities at the HM (i.e., V2 and V3) and hemispheric separation at the VM. Only a few studies have examined FLE during vertical motion and HM crossing. Nagai et al. (2002) tested positions around HM crossing along the VM but pooled data across positions, preventing position-specific analyses. Ichikawa and Masakura (2006, 2010) presented stimuli at 2.5° eccentricity, outside the central range investigated here.

The influence of motion direction relative to retinotopic features has been studied mainly for horizontal motion. Consistent with previous work (Mateeff and Hohnsbein, 1988; Kanai et al., 2004; Shi and Nijhawan, 2008), we observed larger FLE values for VM-petal than VM-fugal motion. Shi and Nijhawan (2008) also reported negative FLE values during VM-fugal motion. Similarly, some participants in our study showed negative or strongly reduced FLE values in VM-fugal conditions (Fig. S3). We speculate that interindividual variability in V1 surface area and retinotopy (Harvey and Dumoulin, 2011) may contribute to these differences. In contrast, studies of vertical motion have focused on absolute direction rather than HM-petal versus HM-fugal motion. Nagai et al. (2002) reported stronger FLE values for downward motion and linked this to representational gravity (Hubbard, 2020), whereas Ichikawa and Masakura (2010) found no directional effect. Our results suggest that such asymmetries are highly localized around the fovea and HM (±0.5°), potentially explaining these discrepancies.

Unlike previous reports (Kanai et al., 2004; Shi and Nijhawan, 2008; Suzuki et al., 2023), we found no significant left-right visual field asymmetry in the FLE (Fig. 2*B*; Fig. S1). However, unlike these reports, our measurements were confined to a narrow central region (±2°). At similar eccentricities, Amadeo et al. (2022) also reported larger FLE values in peripheral than central vision. Altogether, previous studies have largely focused on the relative eccentricity between flash and moving stimuli rather than absolute visual-field position (Baldo et al., 2002; Linares et al., 2007).

### Possible underlying mechanisms

We identified three factors influencing the FLE: crossing retinotopic features, motion direction relative to these features, and eccentricity. Below, we discuss their possible physiological origins.

Crossing retinotopic features involves transitions between distinct cortical representations. This is most evident at the VM for two reasons. First, VM is represented bilaterally through callosal projections restricted to the V1–V2 border (Hubel and Wiesel, 1967; Innocenti et al., 2022) spanning a narrow cortical band equivalent to 0.1–0.2° in central vision (Essen et al., 1982; Kennedy et al., 1986; Kennedy and Dehay, 1988; Clarke and Miklossy, 1990; Zilles and Clarke, 1997).

Second, while horizontal intracortical connections extend over 5–6 mm and conduct relatively slowly (Grinvald et al., 1994; Angelucci et al., 2002; Reynaud et al., 2012; Muller et al., 2014), callosal fibers are ∼10 times longer but myelinated and conduct substantially faster (4.9–11.4 m/s; Caminiti et al., 2009, 2013; Tomasi et al., 2012). Consequently, VM crossing will disrupt the slow horizontal propagation and, according to the PRM hypothesis, should decrease the FLE as reported here and in Benvenuti et al. (2020). Unlike Benvenuti et al., however, we found no effect of traveled distance on FLE amplitude (but see Zhaoping, 2025). A likely explanation is magnification difference between NHP and humans, for which the equivalent visual extent likely covers <1° (Angelucci et al., 2002; Harvey and Dumoulin, 2011), decreasing drastically the scale at which distance-dependent effect should occur.

The HM however represents an anatomical division for visual areas downstream from V1. Unlike V1, upper and lower visual fields are segregated in dorsal and ventral portions of V2 and V3. In contrast to the VM effect, crossing the HM increased the FLE. One possible explanation is that the increase reflects transitions between dorsal and ventral processing streams and involves higher-order retinotopic representations, although the underlying mechanism remains unclear.

The fovea possesses a distinctive cortical organization (Zeki, 1969; Schira et al., 2009), with reduced or atypical retinotopy and patchy feature-selective responses (Qian et al., 2024). The fovea may function as an integrative hub linking visual quadrants, consistent with proposals that central vision operates as a “visual sketchpad” (Zhaoping, 2019; Oletto et al., 2023). Attentional allocation is also enhanced near foveola (Poletti et al., 2017; Poletti, 2023) and is known to reduce the FLE (Baldo and Klein, 1995; Baldo et al., 2002; Sarich et al., 2007; Shioiri et al., 2010). Hence, attention should contribute to the increase of FLE with eccentricity, as observed here. In contrast, this does not explain the larger FLE observed for foveopetal than foveofugal motion. However, the effect of attention was not the purpose of our study, for which a block design ensured spatial stability in the allocated attentional resources (see Materials and Methods). Thus, the contribution of attentional mechanisms to the observed results would require further investigation.

The PRM framework predicts an effect of eccentricity. Because retinocortical magnification decreases with eccentricity (Van Essen et al., 1984; Harvey and Dumoulin, 2011), horizontal connections, a priori of fixed cortical extent, will cover larger regions of visual space and yield to faster propagation in the periphery than in the fovea, when expressed in visual coordinates. This predicts stronger anticipatory effects at larger eccentricities, consistent with our results. A possible confound in the observed effect of eccentricity is that contrast sensitivity decreases with eccentricity (Hilz and Cavonius, 1974; Robson and Graham, 1981; Watson and Ahumada, 2005). However, this modulation is largely explained by cortical magnification, once stimuli are M-scaled (Virsu and Rovamo, 1979; Jigo et al., 2023). Remaining anisotropies across visual-field meridians (Cameron et al., 2002; Abrams et al., 2012; Baldwin et al., 2012) may reflect interactions between retinotopic organization (Himmelberg et al., 2023) and higher-level priors such as representational gravity (Hubbard, 2020), potentially contributing to the asymmetries observed in Experiment 3.

We also observed larger FLE values for petal than fugal motion relative to retinotopic features. Because the same cortical territory is activated in both directions, the critical difference lies in the sequence of activation across retinotopic maps. Cortical magnification factor predicts that cortical activation accelerates when approaching these features and decelerates when moving away. Whether and how this difference would generate opposite FLE modulations remains unclear. Moreover, if directional asymmetries resulted solely from cortical magnification, the FLE should fully recover after crossing into the opposite hemifield. Instead, recovery was only partial, suggesting a further modulation by petal–fugal biases. The switch from horizontal to callosal mediation at the VM may qualitatively alter propagation dynamics, consistent with evidence that callosal deactivation differentially affects VM-petal and VM-fugal responses (Raemaekers et al., 2009; Conde-Ocazionez et al., 2018).

## Conclusion

Using psychophysics, we verified the principal predictions of the PRM hypothesis: retinotopic discontinuities and anisotropies modulate the FLE. The strong reduction associated with VM crossing, together with the absence of a comparable reduction at the HM, suggests that the FLE primarily reflects activity propagation within V1. The eccentricity effect further supports an origin in the early visual cortex (Shipp et al., 1995; Yu et al., 2020; Sedigh-Sarvestani and Fitzpatrick, 2022; Ribeiro et al., 2023). By combining Bayesian adaptive psychophysics with predictions derived from mesoscopic imaging, we show that fine-scale cortical organization can shape perception at a local scale. These results highlight the importance of considering map discontinuities when interpreting psychophysical measurements. Furthermore, the interindividual variability observed here may reflect anatomical variability in retinotopic organization (Harvey and Dumoulin, 2011). Hence, future studies combining psychophysics and neuroimaging should help determine how individual cortical topology shapes perception.
